# Supplementary material for: Optimal minimal residual disease threshold in pediatric acute myeloid leukemia: A retrospective cohort study based on the TARGET database
Source: PLoS Med. 2026 May 8;23(5):e1005088. doi: 10.1371/journal.pmed.1005088 (PMC13155632; doi:10.1371/journal.pmed.1005088)
Supplement: S1 Code — (ZIP) [file pmed.1005088.s002.zip › S2 code/PROJ8_24_tbl/PROJ8_24_tbl.htm]

## ÑÐ¾¿ÈËÈºÃèÊö

|  |  |  |  |  |  |
| --- | --- | --- | --- | --- | --- |
| First Event | No | Yes | Standardize diff. | P-value | P-value\* |
| N | 594 | 611 |  |  |  |
| WBC | 53.8 (77.3) 20.6 (0.6-549.9) | 76.7 (106.0) 35.1 (0.7-918.5) | 0.2 (0.1, 0.4) | <0.001 | <0.001 |
| BM blast(%) | 62.3 (24.2) 68.0 (0.0-100.0) | 66.7 (24.0) 73.0 (0.0-100.0) | 0.2 (0.1, 0.3) | 0.002 | <0.001 |
| Peripheral blasts (%) | 40.7 (30.3) 38.0 (0.0-98.0) | 45.8 (31.7) 46.0 (0.0-99.0) | 0.2 (0.0, 0.3) | 0.005 | 0.005 |
| MRD % at end of course 1 | 0.5 (2.3) 0.0 (0.0-22.0) | 5.6 (15.8) 0.0 (0.0-92.0) | 0.5 (0.3, 0.6) | <0.001 | <0.001 |
| MRD % at end of course 2 | 0.1 (1.0) 0.0 (0.0-12.0) | 3.6 (12.4) 0.0 (0.0-75.0) | 0.4 (0.3, 0.5) | <0.001 | <0.001 |
| Gender |  |  | 0.1 (-0.0, 0.2) | 0.072 | - |
| Male | 292 (49.2%) | 332 (54.3%) |  |  |  |
| Female | 302 (50.8%) | 279 (45.7%) |  |  |  |
| Age group |  |  | 0.0 (-0.1, 0.1) | 0.966 | - |
| £¼10 | 323 (54.4%) | 333 (54.5%) |  |  |  |
| ¡Ý10 | 271 (45.6%) | 278 (45.5%) |  |  |  |
| Chemotherapy Protocol |  |  | 0.1 (-0.1, 0.2) | 0.580 | - |
| AAML1031 | 334 (56.2%) | 335 (54.8%) |  |  |  |
| AAML0531 | 226 (38.0%) | 247 (40.4%) |  |  |  |
| AAML03P1 | 34 (5.7%) | 29 (4.7%) |  |  |  |
| Risk group |  |  | 0.7 (0.5, 0.8) | <0.001 | - |
| Low risk | 327 (56.4%) | 156 (25.8%) |  |  |  |
| Standard risk | 202 (34.8%) | 372 (61.5%) |  |  |  |
| High risk | 51 (8.8%) | 77 (12.7%) |  |  |  |
| Karyotype |  |  | 0.5 (0.4, 0.6) | <0.001 | - |
| MLL | 102 (17.6%) | 154 (25.6%) |  |  |  |
| t(8;21) | 129 (22.3%) | 54 (9.0%) |  |  |  |
| inv(16) | 91 (15.7%) | 55 (9.1%) |  |  |  |
| Normal | 141 (24.4%) | 138 (22.9%) |  |  |  |
| Other | 116 (20.0%) | 201 (33.4%) |  |  |  |
| FLT3-ITD Status |  |  | 0.1 (0.0, 0.2) | 0.028 | - |
| FLT3-ITD-wt | 517 (87.0%) | 504 (82.5%) |  |  |  |
| FLT3-ITD-mt | 77 (13.0%) | 107 (17.5%) |  |  |  |
| NPM1 Status |  |  | 0.3 (0.2, 0.4) | <0.001 | - |
| NPM1-wt | 508 (86.0%) | 578 (94.8%) |  |  |  |
| NPM1-mt | 83 (14.0%) | 32 (5.2%) |  |  |  |
| CEBPA Status |  |  | 0.1 (0.0, 0.3) | 0.016 | - |
| CEBPA-wt | 543 (92.0%) | 581 (95.4%) |  |  |  |
| CEBPA-mt | 47 (8.0%) | 28 (4.6%) |  |  |  |
| WT1 Status |  |  | 0.2 (0.1, 0.4) | 0.006 | - |
| WT1-wt | 246 (95.7%) | 246 (89.5%) |  |  |  |
| WT1-mt | 11 (4.3%) | 29 (10.5%) |  |  |  |
| NUP214 |  |  | 0.0 (-0.1, 0.1) | 0.896 | - |
| Negative | 582 (98.0%) | 598 (97.9%) |  |  |  |
| Positive | 12 (2.0%) | 13 (2.1%) |  |  |  |
| NUP98 |  |  | 0.2 (0.1, 0.3) | <0.001 | - |
| Negative | 587 (98.8%) | 581 (95.1%) |  |  |  |
| Positive | 7 (1.2%) | 30 (4.9%) |  |  |  |
| FAB Category |  |  | 0.4 (0.3, 0.6) | <0.001 | - |
| M0 | 5 (1.1%) | 32 (7.0%) |  |  |  |
| M1 | 54 (12.4%) | 65 (14.3%) |  |  |  |
| M2 | 161 (36.8%) | 104 (22.9%) |  |  |  |
| M4 | 104 (23.8%) | 102 (22.4%) |  |  |  |
| M5 | 80 (18.3%) | 119 (26.2%) |  |  |  |
| M6 | 4 (0.9%) | 6 (1.3%) |  |  |  |
| M7 | 29 (6.6%) | 27 (5.9%) |  |  |  |
| CNS disease |  |  | 0.2 (0.0, 0.4) | 0.046 | - |
| CNS1 | 237 (73.4%) | 214 (66.0%) |  |  |  |
| CNS2 | 68 (21.1%) | 77 (23.8%) |  |  |  |
| CNS3 | 18 (5.6%) | 33 (10.2%) |  |  |  |
| Chloroma |  |  | 0.1 (-0.1, 0.2) | 0.233 | - |
| No | 373 (90.5%) | 381 (88.0%) |  |  |  |
| Yes | 39 (9.5%) | 52 (12.0%) |  |  |  |
| CR status at end of course 1 |  |  | 0.3 (0.2, 0.4) | <0.001 | - |
| CR | 510 (86.3%) | 449 (74.2%) |  |  |  |
| Not in CR | 81 (13.7%) | 156 (25.8%) |  |  |  |
| CR status at end of course 2 |  |  | 0.6 (0.4, 0.7) | <0.001 | <0.001 |
| CR | 587 (100.0%) | 520 (86.2%) |  |  |  |
| Not in CR | 0 (0.0%) | 81 (13.4%) |  |  |  |
| Death | 0 (0.0%) | 2 (0.3%) |  |  |  |
| MRD at end of course 1 |  |  | 0.6 (0.5, 0.7) | <0.001 | - |
| £¼0.1 | 525 (88.4%) | 399 (65.3%) |  |  |  |
| ¡Ý0.1 | 69 (11.6%) | 212 (34.7%) |  |  |  |
| MRD at end of course 2 |  |  | 0.5 (0.3, 0.6) | <0.001 | - |
| £¼0.1 | 571 (96.1%) | 502 (82.2%) |  |  |  |
| ¡Ý0.1 | 23 (3.9%) | 109 (17.8%) |  |  |  |
| SCT in 1st CR |  |  | 0.1 (-0.1, 0.2) | 0.339 | - |
| No | 503 (85.1%) | 440 (83.0%) |  |  |  |
| Yes | 88 (14.9%) | 90 (17.0%) |  |  |  |
| Vital Status |  |  | 1.8 (1.7, 1.9) | <0.001 | - |
| Alive | 593 (99.8%) | 229 (37.5%) |  |  |  |
| Death | 1 (0.2%) | 382 (62.5%) |  |  |  |

±íÖÐ½á¹û:
Mean(SD) Median (Min-Max) / N(%)
PÖµ\*: ÈçÊÇÁ¬Ðø±äÁ¿£¬ÓÃKruskal WallisÖÈºÍ¼ìÑéµÃ³ö, Èç¼ÆÊý±äÁ¿ÓÐÀíÂÛÊý<10£¬ÓÃFisher¾«È·¸ÅÂÊ¼ìÑéµÃ³ö
´Ë±íÓÃÒ×õÍ³¼ÆÈí¼þ (www.empowerstats.com) ºÍRÈí¼þÉú³É£¬Éú³ÉÈÕÆÚ£º 2026-03-21
